# Assessment of Myocardial Iron Overload and Strain Abnormalities in Pediatric β-Thalassemia Using Multiparametric CMR

**DOI:** 10.3390/diagnostics16142139

**Published:** 2026-07-08

**Authors:** Rania Awadi, Narjes Benameur, Mohamed Deriche, Ilhem Ben Fraj, Seif Boukhriba, Aicha Ben Taieb, Monia Ouedreni, Salam Labidi

**Affiliations:** 1 Research Laboratory of Biophysics and Medical Technologies, The Higher Institute of Medical Technologies of Tunis, University of Tunis El Manar, Tunis 1006, Tunisia; rania.awadi@istmt.utm.tn (R.A.); narjes.benameur@istmt.utm.tn (N.B.); salam.labidi@istmt.utm.tn (S.L.); 2 Artificial Intelligence Research Centre, Ajman University, Ajman P.O. Box 346, United Arab Emirates; 3 National Center of Bone Marrow Transplantation, Department of Pediatrics Immunohematology and Stem Cell Transplantation, Faculty of Medicine of Tunis, University of Tunis El Manar, Tunis 1007, Tunisia; ilhem.benfraj@fmt.utm.tn (I.B.F.); aicha.bentaieb@fmt.utm.tn (A.B.T.); monia.ouederni@rns.tn (M.O.); 4 Radiology Department, La Rabta Teaching Hospital, Tunis 1007, Tunisia; seifboukriba@gmail.com

**Keywords:** β-thalassemia major, cardiovascular magnetic resonance, T1 mapping, T2*, artificial intelligence, strain, iron overload

## Abstract

**Background/Objectives:** Myocardial iron overload is a major contributor to adverse cardiac outcomes in pediatric patients with transfusion-dependent β-thalassemia (TDT). Cardiovascular magnetic resonance (CMR), including T2* and T1 mapping, allows quantification of myocardial iron and early detection of cardiac dysfunction. Artificial intelligence (AI)-assisted CMR feature tracking (CMR-FT) provides a sensitive and reproducible approach for assessing myocardial deformation, even in patients with preserved left ventricular ejection fraction (LVEF). This study aimed to evaluate the utility of AI-based CMR-FT and its relationship with multiparametric CMR biomarkers, including myocardial strain (GCS, GLS, GRS), tissue characteristics (T2*, T1), and left ventricular (LV) geometry in pediatric TDT patients. **Methods**: In this retrospective study, 68 pediatric patients with β-thalassemia major and 20 age-matched healthy controls underwent CMR with T2*, T1 mapping, and FT-based strain analysis. Myocardial iron overload was defined as T2* < 20 ms. Strain parameters were compared between groups, and correlations with tissue characteristics and LV geometry were assessed using Pearson’s correlation. **Results:** Patients with myocardial iron overload have significantly reduced GCS compared to controls (−17.4 ± 1.6% vs. −19.3 ± 4.5%, *p* < 0.01). GCS correlated with T2* (r = −0.33, *p* = 0.007) and T1 (r = −0.45, *p* < 0.001). GLS was sensitive to LV geometric changes, particularly concentric remodeling, correlating with LV mass/EDV ratio (r = −0.449, *p* < 0.001). **Conclusions**: AI-based CMR-FT combined with multiparametric tissue imaging enhances early detection of subclinical myocardial dysfunction in pediatric TDT, offering diagnostic insights beyond conventional CMR metrics and supporting improved risk stratification.

## 1. Introduction

β-thalassemia is a severe inherited hemoglobin disorder caused by mutations in the β-globin gene, leading to deficient or absent β-globin chain synthesis. This imbalance results in ineffective erythropoiesis, chronic hemolytic anemia, and a lifelong requirement for blood transfusions, typically initiated early in childhood [1,2,3,4]. While transfusion therapy has markedly improved survival, it inevitably leads to progressive iron overload. Myocardial iron deposition, in particular, remains a major cause of morbidity and mortality, culminating in iron-induced cardiomyopathy and heart failure if not adequately monitored and treated [5,6,7,8]. Accordingly, early identification of cardiac involvement and timely optimization of therapy are essential to improve long-term outcomes in this population.

Cardiovascular magnetic resonance (CMR) is the established non-invasive reference standard for assessing myocardial iron burden and cardiac function [9]. Among available techniques, T2* imaging remains the clinical gold standard for quantifying myocardial iron concentration, with strong evidence demonstrating an inverse relationship between T2* values and tissue iron levels [10,11,12,13,14,15]. However, T2* primarily reflects myocardial iron content and may lack sensitivity in detecting early functional impairment before the onset of overt ventricular dysfunction [15,16,17]. In this context, CMR Feature Tracking (CMR-FT) has emerged as a robust technique for quantifying myocardial deformation directly from standard cine images. CMR-FT-derived strain parameters have demonstrated high sensitivity for detecting subclinical myocardial dysfunction in patients with β-thalassemia, even when left ventricular ejection fraction (LVEF) remains preserved [14,18]. In transfusion-dependent thalassemia (TDT), progressive myocardial iron accumulation induces subtle alterations in myocardial mechanics that may not be captured by conventional functional indices but can be detected through early changes in global strain parameters [19]. Recent advances in artificial intelligence (AI), particularly deep learning, have further transformed cardiovascular imaging analysis by enabling automated, reproducible, and efficient quantification of myocardial function. Automated myocardial strain analysis reduces inter-observer variability and improves measurement consistency compared to manual or semi-automated approaches [20]. Several recent studies have demonstrated the potential of AI-based strain analysis across different imaging modalities and cardiac diseases. For instance, Ghadimi et al. [21] introduces a fully automated deep learning pipeline for global and segmental circumferential strain analysis from cine displacement encoding with stimulated echoes (DENSE) CMR, eliminating manual segmentation and phase unwrapping. In another study [22], researchers developed Motion-Echo, a deep learning framework trained on over 11,000 pediatric echocardiograms, automates myocardial strain analysis to create standardized “digital profiles” of children’s hearts, enabling earlier detection of cardiac dysfunction with high precision across vendors and image qualities. A recent study by Abramikas et al. [23] evaluated the performance of an AI-based CMR feature tracking tool for the assessment of LV global and segmental GLS in Aortic stenosis patients using SuiteHEART software (version 5.1.1, Neosoft, Pewaukee, WI, USA) provides accurate global longitudinal strain (GLS).

Beyond conventional T2* assessment, novel MRI biomarkers have been increasingly investigated to provide a more comprehensive evaluation of iron overload cardiomyopathy. Native T1 mapping has been shown to reflect diffuse myocardial tissue alterations and may detect iron-related changes even in cases where T2* values are only mildly abnormal [24,25]. In addition, strain imaging parameters and left ventricular geometric indices, such as LV mass and wall thickness, provide complementary information on myocardial remodeling and mechanical adaptation [26,27]. These multiparametric biomarkers have been associated with early myocardial dysfunction and may improve risk stratification when used in combination rather than in isolation. Accordingly, multiparametric CMR approaches integrating tissue characterization, myocardial deformation, and structural assessment are increasingly considered more informative than single-parameter evaluation strategies [28,29,30,31].

Despite these advances, several important limitations remain in the current literature. First, AI-based CMR feature tracking tools have shown promising results; however, their application in pediatric transfusion-dependent β-thalassemia populations remains limited and insufficiently validated. Second, most existing studies have evaluated myocardial strain, tissue characterization, or geometric parameters independently rather than within an integrated multiparametric framework. Third, strain assessment has often been restricted to single global indices, without comprehensive evaluation of multiparametric myocardial deformation. Finally, the incremental value of combining myocardial deformation, iron quantification, and left ventricular geometry for early risk stratification has not yet been fully established. The scientific problem addressed in this study is the limited validation of AI-based CMR feature tracking (CMR-FT) for early detection of cardiac involvement in pediatric β-thalassemia, as well as the lack of an integrated multiparametric approach combining myocardial deformation, iron quantification, and ventricular remodeling.

In this context, the present study aims to evaluate the utility of AI-based CMR feature tracking (CMR-FT) for the early detection of cardiac involvement in pediatric patients with TDT. Specifically, we investigate the relationships between global myocardial strain and key CMR-derived parameters, including T2*, native T1 mapping, and left ventricular (LV) geometric indices.

The main contributions of this study are as follows:

- We evaluate the utility of AI-based CMR feature tracking (CMR-FT) for detecting subclinical cardiac involvement in pediatric transfusion-dependent β-thalassemia.
- We integrate AI-based CMR-FT–derived strain (GLS, GCS, GRS) with T2* and native T1 mapping in a pediatric cohort.
- We show that GCS is more closely related to myocardial tissue characteristics, while GLS reflects early left ventricular remodeling.
- We analyze 68 patients and 20 age-matched controls, representing one of the largest pediatric CMR datasets in this field.

## 2. Materials and Methods

**Figure 1:**
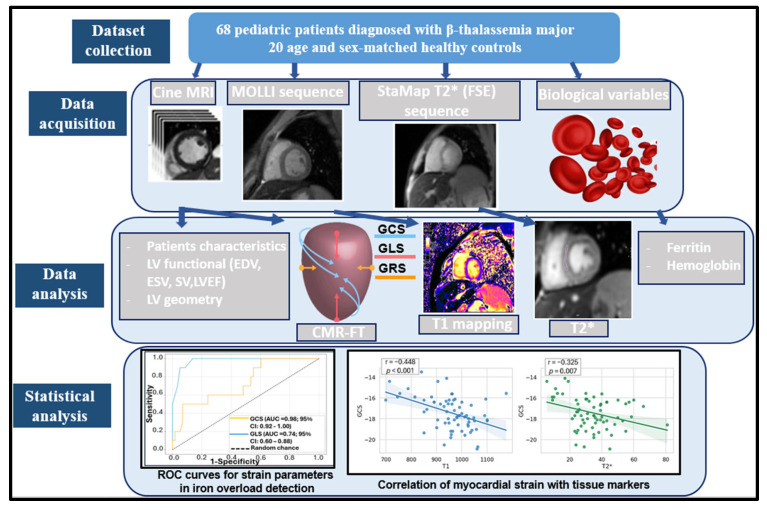
Proposed framework for multiparametric CMR analysis in patients with β-thalassemia major.

The proposed multiparametric CMR framework for the evaluation of patients with β-thalassemia major is summarized in Figure 1.

Briefly, the workflow comprised three steps: (1) CMR data collection, (2) image processing and quantitative analysis, including T2*, T1 mapping, and AI-based CMR-FT strain assessment, and (3) multiparametric integration to evaluate the relationships between myocardial iron overload, strain indices, and LV geometry.

### 2.1. Study Population

This retrospective study included 68 pediatric patients with a confirmed diagnosis of β-thalassemia major who were followed at the Department of Pediatric Hematology and Immunology, National Bone Marrow Transplant Center, Tunis. All patients underwent clinically indicated cardiac and hepatic MRI examinations at the Radiology Department of La Rabta University Hospital between 2021 and 2022. The study protocol was approved by the institutional ethics committee of the National Bone Marrow Transplant Center, and all procedures were conducted in accordance with established ethical standards for research involving human subjects. The inclusion criteria are as follows: (i) confirmed diagnosis of β-thalassemia major based on hemoglobin electrophoresis, (ii) transfusion dependency, (iii) age between 5 and 18 years, and (iv) availability of a complete CMR examination including cine imaging, T1 mapping, and T2* sequences. Patients were required to have no contraindications to MRI, including cardiac implantable electronic devices, ferromagnetic implants, or severe claustrophobia.

**Figure 2:**
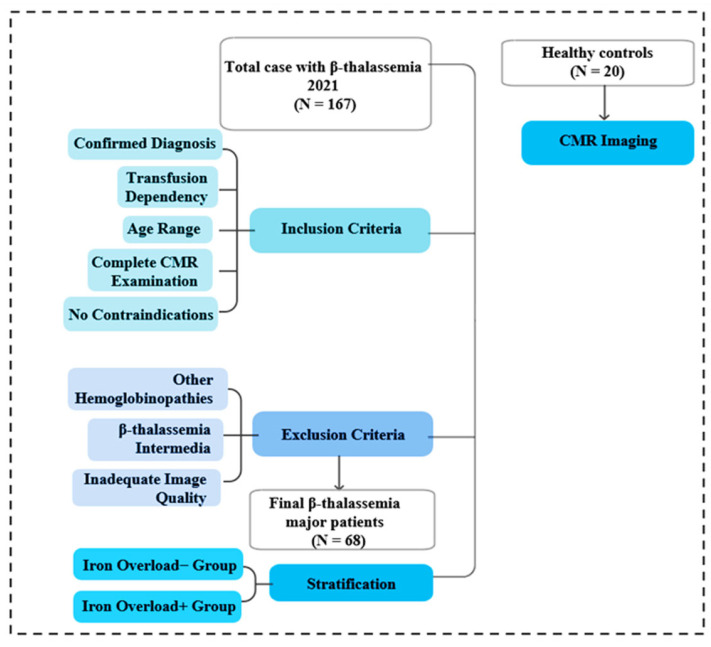
Flowchart of study population selection.

The exclusion criteria are as follows: (i) patients with other transfusion-dependent hemoglobinopathies (e.g., sickle cell disease) or rare hematological disorders were excluded, (ii) patients with β-thalassemia intermedia, due to differences in transfusion burden and cardiac involvement patterns, (iii) examinations with inadequate image quality, including those affected by significant motion artifacts or poor breath-holding compromising post-processing (Figure 2).

Based on myocardial T2* measurements, patients were stratified into two groups:

- Iron overload− group: no evidence of myocardial iron accumulation (normal T2* values).
- Iron overload+ group: presence of myocardial iron overload (reduced T2* values).

Additionally, 20 healthy individuals matched for age and sex, without cardiac symptoms or any history of cardiac disease, were included as controls.

### 2.2. CMR Acquisition and Analysis

CMR scans were performed using 1.5 Tesla system (SIGNA Artist, GE Healthcare) with ECG gating. Cardiac localization was initially achieved using a non-gated scout sequence, followed by acquisition of standard long-axis and short-axis views, including two-, three-, and four-chamber views as well as contiguous short-axis slices covering the entire LV. Cine images were acquired using a balanced steady-state free precession (bSSFP) sequence with the following parameters: repetition time (TR) = 4 ms, echo time (TE) = 1 ms, flip angle = 60°, matrix size = 256 × 256, slice thickness = 8 mm, and inter-slice gap = 1 mm. Temporal resolution ranged from 20 to 50 ms, enabling accurate assessment of cardiac motion throughout the cardiac cycle.

Native T1 mapping was conducted using a Modified Look-Locker Inversion Recovery (MOLLI) sequence acquired during diastole at three LV levels (base, mid-ventricle, and apical). Acquisition parameters included the following: TR = 3.6 ms, TE = 1.6 ms, slice thickness = 10 mm, inter-slice gap = 13 mm, flip angle = 35°, matrix size = 190 × 140, voxel size = 1.9 × 2.6 mm^2^, and acceleration factor = 2. Myocardial T2* imaging was performed using a multi-echo gradient or fast spin echo sequence (StaMap T2*), with a TR of 20 ms, TE = 8 ms, slice thickness = 10 mm, inter-slice gap = 18 mm, and flip angle = 20°. Three short-axis slices (basal, mid-ventricular, and apical) were acquired during diastole.

All CMR datasets were analyzed using commercially available software (CVI42, version 6.0.2, Circle Cardiovascular Imaging, Calgary, AB, Canada). Left ventricular endocardial and epicardial contours were initially delineated using a deep learning-based automated segmentation algorithm based on convolutional neural network (CNN), applied to both short-axis and long-axis cine images. All automatically generated contours were systematically reviewed and manually adjusted when necessary by an experienced operator to ensure anatomical accuracy. Based on these contours, key LV functional and geometric parameters were calculated, including LVEF, end-diastolic volume (EDV), end-systolic volume (ESV) and left ventricle mass (LVM).

**Figure 3:**
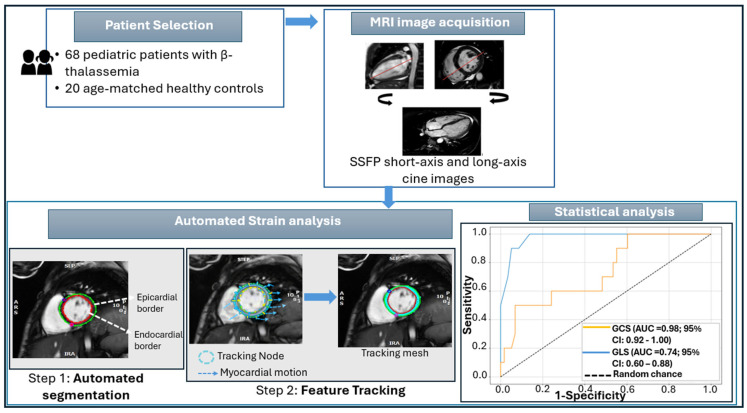
Schematic representation of the myocardial strain analysis process.

**Figure 4:**
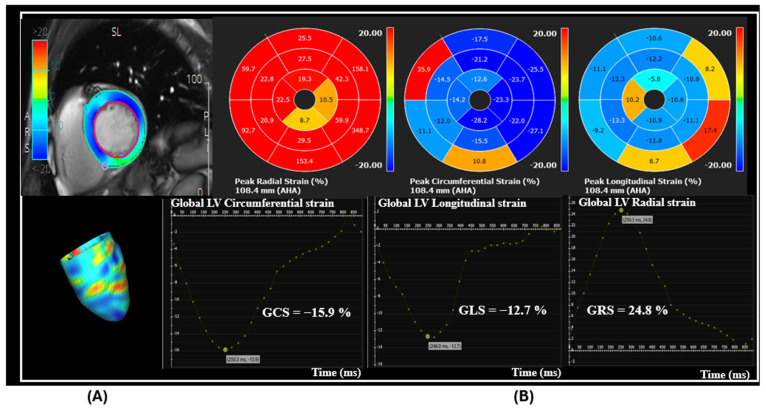
Global myocardial strain assessment in a patient with β-thalassemia. (**A**) Short-axis CMR image showing endocardial and epicardial contours with three-dimensional myocardial strain mapping. (**B**) Corresponding strain curves derived from CMR-FT.

Once the endocardial border is automatically delineated, it is propagated throughout the cardiac cycle using the FT algorithm based on non-rigid registration and optical flow techniques (Figure 3). This approach tracks myocardial motion by following pixel intensity patterns along the endocardial and epicardial contours and estimating frame-by-frame displacements within regions of interest. Deformation is quantified using virtual tracking points (imaginary nodes) placed along the midline between both borders, which move according to the computed motion field throughout the cardiac cycle. Global strain parameters were then derived, including global longitudinal strain (GLS) from long-axis views and global circumferential (GCS) and radial strain (GRS) from short-axis mid-ventricular images (Figure 4).

**Figure 5:**
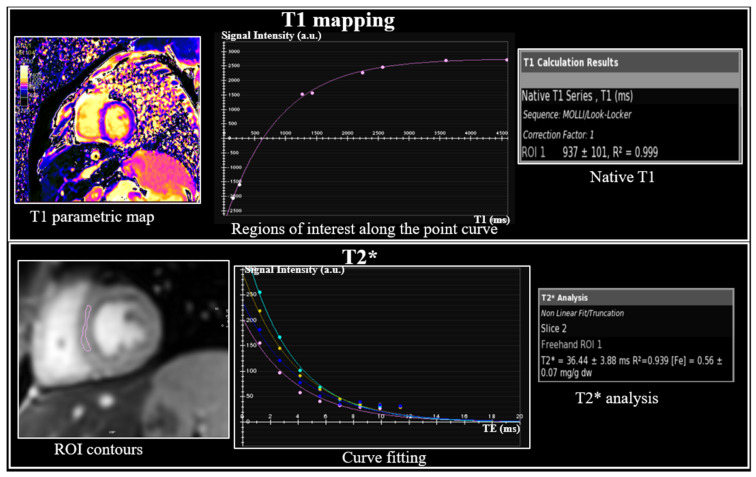
T1 mapping and T2* analysis in a patient with β-thalassemia and myocardial iron overload. Top: Native T1 parametric map with corresponding curve and quantitative analysis, demonstrating a myocardial T1 value of 937 ms. Bottom: Short-axis CMR image with T2 analysis, showing a T2* value of 36.44 ms in the same patient.

Myocardial T2* values were quantified by analyzing signal intensity decay across multiple echo times. Regions of interest (ROIs) were consistently placed in the interventricular septum to minimize susceptibility artifacts and ensure reproducibility. Myocardial iron overload was defined as T2* < 20 ms, whereas normal iron levels were defined as T2* ≥ 20 ms [32]. For T1 mapping, ROIs were carefully placed within the myocardium, avoiding blood pool and epicardial fat. Pixel-wise relaxation curves were generated automatically, along with goodness-of-fit indicators (R^2^ values) to ensure measurement reliability. Subsequently, parametric T1 maps were produced for both qualitative and quantitative assessment (Figure 5). Native T1 values > 998 ms were considered normal, whereas values ≤ 998 ms were considered abnormal, suggesting myocardial involvement.

### 2.3. Statistical Analysis

Statistical analyses were performed using SPSS software (version 23.0; IBM Corp., Armonk, NY, USA). Continuous variables were tested for normality and are presented as mean ± standard deviation when normally distributed. Group comparisons were performed using the independent samples Student’s *t*-test. Correlations between variables were assessed using Pearson’s correlation coefficient for normally distributed data and Spearman’s rank correlation coefficient otherwise.

Multivariable linear regression analysis was conducted to evaluate independent associations between myocardial strain parameters and tissue characteristics. Reproducibility analysis was performed in a randomly selected subset of 10 cases. Intra-observer and inter-observer agreement for GLS, GCS, and GRS measurements were assessed using intraclass correlation coefficients (ICC) with corresponding 95% confidence intervals. A two-sided *p*-value < 0.05 was considered statistically significant.

## 3. Results

### 3.1. Descriptive Characteristics of Enrolled Subjects

The descriptive characteristics of the study population comprising 68 patients with β-thalassemia (38 males, 30 females) and 20 healthy controls (17 males, 3 females) are summarized in Table 1. The average age in β-thalassemia patients was 10.78 ± 3.12 years (range 5–17), compared to 11.30 ± 2.72 years (range 5–16) within healthy controls. Significant variations were noted in both height and body surface area (BSA). Patients with β-thalassemia were significantly shorter (136.07 ± 16.00 cm) than the controls (145.65 ± 14.77 cm, *p* = 0.019) and had a significantly lower BSA (1.14 ± 0.22 m^2^ vs. 1.34 ± 0.25 m^2^, *p* < 0.001). Although the average body mass index (BMI) was slightly lower in the β-thalassemia group (18.09 ± 3.53 kg/m^2^) as compared with healthy controls (19.26 ± 3.23 kg/m^2^), though the difference did not reach statistical significance (*p* = 0.189). Similarly, no significant difference was found in heart rate (HR) among both groups (*p* = 0.190). As expected, ferritin and hemoglobin (Hbg) levels were reported only for patients with β-thalassemia. These patients exhibited markedly elevated ferritin levels 1759.68 ± 1210.83 μg/mL (range 129 to 5323 μg/mL) and significantly reduced hemoglobin levels 7.75 ± 0.78 g/dL (range 5.6 to 9.5 g/dL), consistent with chronic anemia characteristic of the disease.

### 3.2. LV Functional and Geometry Measures

LV functional and geometry parameters based on CMR imaging, including ESV, EDV, SV, LVEF, LVM, and the LVM/EDV ratio are summarized in Table 2. Patients with β-thalassemia showed significantly higher indexed LVESV (40.93 ± 12.92 mL/m^2^ vs. 30.87 ± 4.69 mL/m^2^) and LVEDV (101.70 ± 21.47 mL/m^2^ vs. 80.09 ± 6.80 mL/m^2^) compared to healthy controls. Similarly, LVSV was elevated in the β-thalassemia group (60.90 ± 14.19 vs. 48.47 ± 7.06 mL/m^2^), all differences are statistically significant (*p* < 0.001).

Despite these volumetric changes, no statistically significant variation in LVEF was observed between the groups (60.37 ± 5.81% in the β-thalassemia group vs. 61.60 ± 4.66% in controls; *p* = 0.390). However, LVM was significantly higher in the β-thalassemia patients (62.38 ± 14.41 vs. 40.20 ± 5.25 g/m^2^, *p* < 0.001). This disproportionate increase in mass relative to volume was further reflected by a significantly elevated LVM/EDV ratio (0.62 ± 0.10 vs. 0.50 ± 0.13; *p* < 0.001).

### 3.3. Global Myocardial Strain

The study compared global myocardial strain parameters GCS, GLS, and GRS, between β-thalassemia patients and healthy controls. The results, as well as their range and 95% confidence intervals, are presented in Table 3. The mean GCS in β-thalassemia patients was −17.4 ± 1.6% (95% CI: −17.8 to −16.9), which was markedly reduced compared to controls (−19.3 ± 4.5%, 95% CI: −20.0 to −18.7). Similarly, GLS showed notable dysfunction in β-thalassemia patients (−14.7 ± 1.6%, 95% CI: −15.1 to −14.4) compared to controls (−17.1 ± 5.9%, 95% CI: −17.6 to −16.7). Conversely, GRS was markedly decreased in β-thalassemia patients (28.6 ± 3.1%, 95% CI: 27.9 to 29.4) than in controls (31.7 ± 2.5%, 95% CI: 30.6 to 32.8), all *p* < 0.001.

### 3.4. Comparison Strain and Mapping in β-Thalassemia

**Figure 6:**
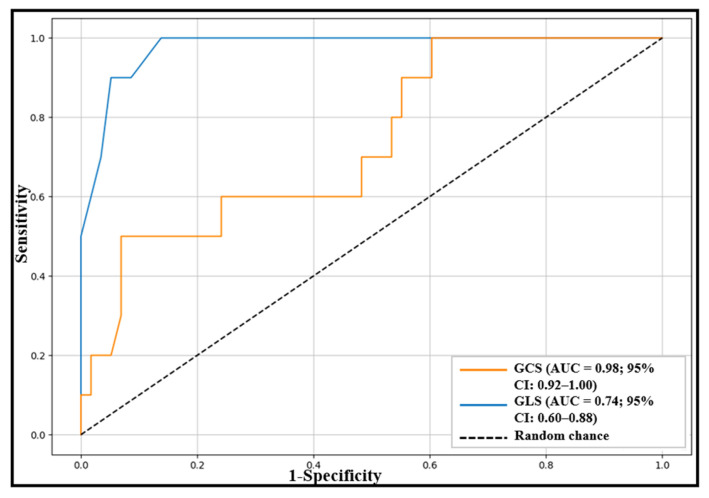
ROC curves demonstrating the diagnostic performance of GCS and GLS for distinguishing β-thalassemia patients with and without myocardial iron overload.

Among the total 68 β-thalassemia patients, 10 individuals (14.7%) were identified with myocardial iron overload, characterized by a T2* value < 20 ms. Table 4 presents a comparison of cardiac MRI strain, T1 mapping, and T2* measurements across both groups. Patients presenting with elevated iron overload exhibited significantly reduced myocardial strain compared to those without overload. Figure 6 illustrates the diagnostic accuracy of both GCS and GLS for detecting iron overload in β-thalassemia patients via ROC curve analysis in both groups. GCS demonstrated significant diagnostic performance, with an area under the curve (AUC) of 0.98 (95% CI: 0.92–1.00), indicating near-perfect accuracy. It achieved 100% sensitivity, meaning it correctly identified all true positive cases, and a specificity of 86.2%, reflecting a strong ability to correctly rule out negative cases. Notably, only 10 patients had myocardial iron overload, which may contribute to the high observed AUC (0.98) and sensitivity of 100% for GCS. Bootstrap analysis confirmed a robust AUC of 0.975 (95% CI: 0.92–1.00); however, the small sample size limits statistical precision, and wider confidence intervals should be expected in larger cohorts.

In contrast, the GLS showed moderate performance, with an AUC of 0.74 with a 95% CI of 0.60–0.88. It had a lower sensitivity of 50.0%, suggesting it missed half of the true positives, but a high specificity of 93.1%, indicating it was effective in identifying true negatives. T1 mapping values were markedly decreased in patients with iron overload 816.0 ± 93.5 ms (range 699 to 962 ms) compared to those without iron overload 980.9 ± 68.7 ms (range 850 to 1175 ms). Similarly, myocardial T2* values were also markedly reduced in the iron overload group 12.2 ± 4.1 ms (range 5.6 to 19.0 ms) versus the non-overload group 37.7 ± 12.2 ms (range 21.5 to 81.0 ms) all *p*-value < 0.001.

### 3.5. Correlation Between Global Myocardium Strain and Tissue Markers, Biological Variables, and Geometric Parameters

**Figure 7:**
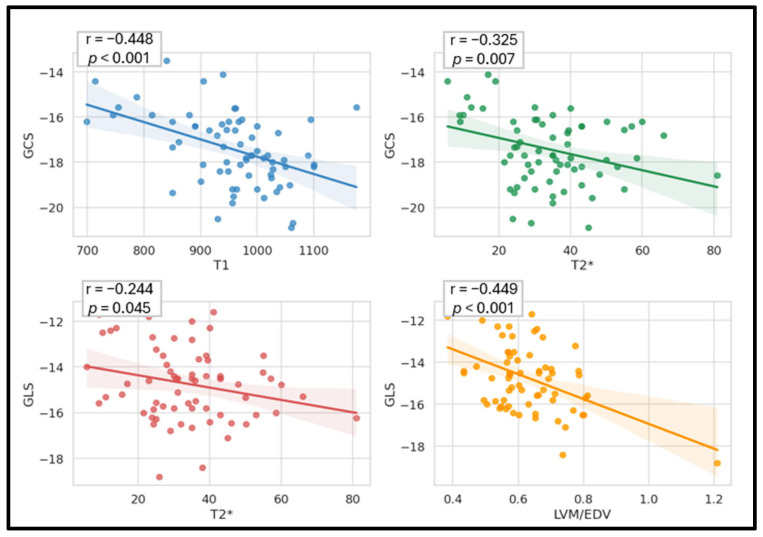
Linear correlation between global myocardial strain, tissue markers and LVM/EDV in patients with β-thalassemia.

Table 5 presents the correlations between global myocardial strain and various tissue markers, biological variables, and geometric parameters across the entire cohort of 68 pediatric patients with β-thalassemia. Among tissue markers, T2* values demonstrated a meaningful inverse correlation with both GCS (r = –0.325, 95% CI [–0.53, –0,09], *p* = 0.007) and GLS (r = –0.244, 95% CI [–0.46, –0.00], *p* = 0.045) as illustrated Figure 7. However, no significant association was observed between T2* values and GRS. T1 mapping values exhibited a negative correlation with GCS (r = –0.448, 95% CI [–0.62, –0,23], *p* < 0.001) and a significant positive association with GRS (r = 0.283, 95% CI [0.05, 0.49], *p* = 0.019).

In contrast, no significant association was found with GLS (*p* = 0.307). Among the biological variables, ferritin showed a very weak positive association with GLS (r = 0.025), which did not reach statistical significance (*p* = 0.840). Similarly, hemoglobin (Hgb) levels did not exhibit any significant correlations with strain parameters (all *p*-values > 0.05). Regarding cardiac geometric parameters, LVM demonstrated a positive, though not statistically significant, correlation with GCS (r = 0.204, *p* = 0.095), and a statistically significant negative correlation with GLS (r = –0.163, 95% CI [−0.38, −0.01], *p* = 0.041). Notably, the LVM/EDV showed a moderate inverse association with GLS (r = –0.449, 95% CI [–0.62, –0.23], *p* < 0.001) but was not significantly associated with either GCS or global radial strain (GRS) (*p* > 0.05 for both).

### 3.6. Multivariable Linear Regression Analysis

Multivariable linear regression was performed with myocardial strain parameters as the dependent variables and myocardial T2* as the main independent variable, adjusting for BSA, hemoglobin level, LVM, age, and sex. Myocardial T2* remained significantly associated with GCS (β = –0.281, *p* = 0.021) and GLS (β = –0.263, *p* = 0.034), indicating an independent effect of myocardial iron overload on these strain parameters. No significant association was observed with GRS was not statistically significant (β = 0.054, *p* = 0.723) as shown in Table 6.

### 3.7. Reproducibility Analysis of Strain Measurements

The reproducibility of myocardial strain measurements was assessed by calculating intraclass correlation coefficients (ICC) for both intra- and inter-observer analyses (Table 7). Intra-observer reproducibility was excellent for GCS (ICC = 0.99) and GLS (ICC = 0.97), while GRS demonstrated good reproducibility (ICC = 0.82). Inter-observer reproducibility was evaluated by an independent second observer repeating all measurements, yielding ICC values indicative of excellent agreement: GCS, 0.90; GLS, 0.98; and GRS, 0.91. These results confirm that CMR-FT–derived strain metrics are highly reliable, supporting their use for quantitative assessment of subclinical myocardial dysfunction.

## 4. Discussion

In this study, we conducted a retrospective analysis on cardiac iron overload in transfusion-dependent patients diagnosed with β-thalassemia major using CMR imaging, with a particular focus on global myocardial strain analysis, tissue markers, biological variables, and geometric parameters. This study yielded several notable findings. First, automated global myocardial strain assessed by CMR-FT was significantly impaired in β-thalassemia patients, including those with preserved LVEF and no evidence of myocardial iron overload. Second, multiparametric analysis incorporating CMR-FT strain, native T1 mapping, and T2* imaging revealed significant correlations among these parameters, highlighting their complementary roles in detecting myocardial iron deposition. Third, increased concentric remodeling, as reflected by the LVM/EDV ratio, was significantly associated with impaired GLS, suggesting that longitudinal strain is particularly sensitive to early geometric changes.

β-thalassemia is characterized by defective hemoglobin synthesis leading to severe anemia [33]. Regular transfusions are necessary to manage anemia but result in progressive iron accumulation, particularly in the heart and liver, significantly increasing the risk of organ damage and mortality [32,34]. Non-invasive assessment of myocardial iron using CMR-based techniques, such as T2* and T1 mapping, is well-established [16,35,36]. CMR-FT, derived strain analysis has emerged as a sensitive tool for detecting subclinical myocardial dysfunction, offering incremental value for early diagnosis and comprehensive cardiac assessment [37,38].

Consistent with prior studies, we observed a significant reduction in myocardial strain in β-thalassemia patients compared to healthy controls. Strain impairment was more pronounced among patients with myocardial iron overload (T2* < 20 ms) than in those without (T2* ≥ 20 ms). For instance, GCS, GLS, and GRS were all significantly reduced in iron-overloaded patients, with GCS showing the largest difference, suggesting that circumferential strain may serve as the most sensitive marker of myocardial remodeling in this context. Similar findings were reported by Rezaeian et al. [28] in a cohort of 91 β-thalassemia patients (66 with myocardial iron overload and 25 without). They observed significantly reduced strain values in patients with myocardial iron overload: GCS −13.57 ± 10.26% vs. −17.44 ± 2.38%, GLS −12.90 ± 4.18% vs. −13.81 ± 3.25%, and GRS 36.7 ± 18.82% vs. 40.50 ± 8.93%. Our cohort yielded comparable results: GCS −15.07 ± 1.09% vs. −17.82 ± 1.63%, GLS −13.52 ± 1.67% vs. −14.95 ± 1.53%, and GRS 25.28 ± 3.04% vs. 29.19 ± 2.73%. Among these parameters, GCS showed the greatest difference between groups, supporting its utility as the most sensitive indicator of myocardial strain impairment and global myocardial remodeling in the presence of iron overload. In contrast, GLS remained a sensitive marker of early myocardial injury, while GRS provided complementary information on global ventricular contractile performance. Combined assessment of these strain parameters enables a more comprehensive evaluation of myocardial dysfunction. ROC analysis further confirmed the diagnostic value of GCS highlighting its superior ability to detect cardiac iron overload.

T2* values were significantly and negatively correlated with both GCS and GLS, whereas no significant correlation was observed with GRS. These findings are consistent with Parsaee et al. [29], who reported a significant correlation between GLS assessed by speckle-tracking echocardiography and T2* values, reinforcing the link between impaired myocardial deformation and iron overload. Similarly, Meloni et al. [30] reported that higher myocardial iron concentrations were associated with worsening GLS. Ansah et al. [31] further identified a correlation between GCS and T2*, supporting the association between myocardial iron overload and impaired strain. Baseline T1 mapping values were negatively associated with GCS and positively correlated with GRS, but showed no meaningful association with GLS. This aligns with previous observations by Ojha et al. [16], who reported reduced T1 values in β-thalassemia patients correlated with impaired GRS, an early indicator of myocardial involvement.

Although ferritin is commonly used as a secondary indicator of overall body iron and is typically elevated in TDT patients [2], we found no correlation between ferritin levels and global myocardial strain parameters. This finding aligns with existing literature, which reports a weak association between serum ferritin and cardiac T2* values [32,36], underscoring the limitations of ferritin as a reliable surrogate of myocardial iron.

Regarding LV geometry, we observed a significant correlation between the LVM/EDV ratio, a marker of concentric remodeling and GLS, whereas no significant associations were observed with GCS or GRS. This is in agreement with Zhang et al. [27], demonstrated that concentric LV remodeling predominantly affects longitudinal strain, while circumferential and radial strain remain relatively preserved. Similar patterns have been described in early-stage hypertrophic cardiomyopathy, where reductions in GCS, GLS, and GRS reflect global myocardial dysfunction [39].

CMR-FT is typically a semi-automated technique based on contour propagation throughout the cardiac cycle [40]. In this study, we evaluated the utility of an AI-based CMR-FT approach in a pediatric population with β-thalassemia, addressing an important gap in the application of strain analysis in pathological myocardium. Our findings support the feasibility of AI-assisted strain analysis in clinical practice, provided that careful quality control of segmentation is performed. This highlights the importance of integrating human supervision with automated tools to ensure reliable quantification in pathological conditions. These results are consistent with previous studies [21,22,23], which have demonstrated the potential of AI-based strain analysis across different imaging modalities and a range of cardiac diseases, thereby reinforcing its broader applicability in clinical cardiovascular imaging.

Overall, this study contributes to the growing evidence supporting AI-based CMR-FT as a clinically applicable tool for the early detection of myocardial dysfunction in pediatric cardiovascular disease. It also highlights the clinical utility of a multiparametric CMR approach integrating strain analysis with T1 and T2* mapping for early risk stratification, timely detection of myocardial involvement, and potential optimization of chelation therapy.

Several limitations should be acknowledged. This was a single-center study, which may limit generalizability. The relatively small number of patients with confirmed cardiac iron overload may have reduced statistical power for subgroup analyses. Additionally, relevant clinical variables, such as transfusion burden, chelation therapy status, and disease duration, were not consistently available, limiting the ability to fully adjust for potential confounders. Despite these limitations, the study provides important insights into the early detection of subclinical myocardial dysfunction in pediatric β-thalassemia. Future studies should aim to include larger, multicenter cohorts, incorporate additional clinical variables, and explore advanced AI-based CMR analysis to further improve early detection and personalized management of cardiac iron overload.

## 5. Conclusions

Our study demonstrates the utility of AI-based CMR feature tracking for the assessment of global strain. Particularly, global circumferential strain (GCS) is significantly impaired in pediatric patients with myocardial iron overload and exhibits strong correlations with myocardial tissue characteristics assessed by T1 mapping and T2*. GLS, in contrast, appears especially sensitive to left ventricular geometric changes, including concentric remodeling, reflecting early structural and functional adaptations that may precede overt systolic dysfunction.

These findings emphasize the added diagnostic value of integrating AI-based CMR–FT derived strain analysis with T1 mapping and T2* imaging. This multiparametric approach enables earlier detection of subclinical myocardial dysfunction and provides a more comprehensive assessment of cardiac involvement in TDT patients, potentially improving disease monitoring and clinical decision-making.

## Figures and Tables

**Table 1:** Descriptive characteristics of the study population.

Characteristics	β-Thalassemia (*N* = 68)	Controls (*N* = 20)	*p*-Value
Age (year)	10.78 ± 3.12	11.30 ± 2.72	0.397
Sexe (M/F)	38/30	17/3	-
Height (cm)	136.07 ± 16.00	145.65 ± 14.77	**0.019**
BMI (kg/m^2^)	18.09 ± 3.53	19.26 ± 3.23	0.189
BSA (m^2^)	1.14 ± 0.22	1.34 ± 0.25	**<0.001**
HR (bpm)	85.30 ± 12.53	89.30 ± 9.52	0.190
Ferritin(μg/L)	1759.68 ± 1210.83	-	-
Hbg (g/dL)	7.75 ± 0.78	-	-

Data are presented as means ± SD. M, male; F, female; BMI, body mass index; BSA, body surface area; HR, heart rate; Hbg, hemoglobin. Significant *p*-values are indicated in bold.

**Table 2:** Left ventricle functional and geometric metrics of patients with β-thalassemia (*N* = 68) VS controls (*N* = 20).

Parameter	β-Thalassemia (*N* = 68)			Controls (*N* = 20)			*p*-Value
	Mean ± SD	Range	95% CI	Mean ± SD	Range	95% CI	
LVESV (mL/m^2^)	40.93 ± 12.92	17–75	37.80–44.06	30.87 ± 4.69	22–40	28.68–33.06	**0.001**
LVEDV (mL/m^2^)	101.70 ± 21.47	38–182	96.50–106.89	80.09 ± 6.80	70–99	76.91–83.28	**<0.001**
LVSV (mL/m^2^)	60.90 ± 14.19	21–107	45.17–51.78	48.47 ± 7.06	40–62	57.47–64.34	**<0.001**
LVEF (%)	60.37 ± 5.81	42–72	58.97–61.78	61.60 ± 4.66	53–72	59.41–63.78	0.390
LVM (g/m^2^)	62.38 ± 14.41	28–116	58.89–65.87	40.20 ± 5.25	26–48	37.74–42.66	**<0.001**
LVM/EDV(g/mL)	0.62 ± 0.10	0.38–1.02	0.59–0.65	0.50 ± 0.13	0.35–0.59	0.47–0.53	**<0.001**

Data are presented as mean ± SD. Confidence intervals (CI) are provided where applicable. Volumes were normalized to BSA using the Boyd equation. Abbreviations: LVESV, left ventricle end-systolic volume; LVEDV, left ventricle end-diastolic volume; LVSV, left ventricle stroke volume; LVEF, left ventricle ejection fraction; LVM, left ventricle mass; EDV, end-diastolic volume. Significant *p*-values are indicated in bold.

**Table 3:** Global myocardial strain of study subjects.

	β-Thalassemia (*N* = 68)			Controls (*N* = 20)			*p*-Value
	Mean ± SD	Range	95% CI	Mean ± SD	Range	95% CI	
GCS (%)	−17.4 ± 1.6	−20.9 to −13.5	−17.8 to −16.9	−19.3 ± 4.5	−21.7 to −16.1	−20.0 to −18.7	**<0.001**
GLS (%)	−14.7 ± 1.6	−18.8 to −11.5	−15.1 to −14.4	−17.1 ± 5.9	−18.4 to −15.3	−17.6 to −16.7	**<0.001**
GRS (%)	28.6 ± 3.0	21.3 to 34.3	27.9 to 29.4	31.7 ± 2.5	25.9 to 35.0	30.6 to 32.8	**<0.001**

GCS, global circumferential strain; GLS, global longitudinal strain; GRS, global radial strain. Confidence intervals (CI) are provided where applicable. Significant *p*-values are indicated in bold.

**Table 4:** Cardiac MRI strain and mapping in β-thalassemia with and without myocardial iron overload.

Parameter	Total *N* = 68	Iron Overload− *N* = 58			Iron Overload+ *N* = 10			*p*-Value
	Mean ± SD	Mean ± SD	Range	95% CI	Mean ± SD	Range	95% CI	
GCS (%)	−17.4 ± 1.6	−17.8 ± 1.6	−20.9 to −15.6	−18.2 to −17.5	−15.1 ± 1.1	−16.2 to −13.5	−15.7 to −14.4	**<0.001**
GLS (%)	−14.7 ± 1.6	−14.9 ± 1.5	−18.8 to −11.6	−15.4 to −14.6	−13.5 ± 1.7	−15.6 to −11.5	−14.7 to −12.4	**0.009**
GRS (%)	28.6 ± 3.1	29.2 ± 2.7	22.4 to 34.3	28.5 to 29.9	25.3 ± 3.0	21.3 to 29	23.1 to 27.5	**<0.001**
T1 (ms)	956.7 ± 93.0	980.9 ± 68.7	850 to 1175	962.9 to 998.9	816.0 ± 93.5	699 to 962	749.1 to 882.9	**<0.001**
T2* (ms)	33.9 ± 14.5	37.7 ± 12.2	21.5 to 81.0	34.5 to 40.9	12.2 ± 4.1	5.6 to 19.0	9.3 to 15.1	**<0.001**

GCS, global circumferential strain; GLS, global longitudinal strain; GRS, global radial strain. Significant *p*-values are indicated in bold.

**Table 5:** Correlation between global myocardium strain and tissue markers, biological variables and geometric parameters.

Variable	GCS (%)		GLS (%)		GRS (%)	
	r	*p*-Value	r	*p*-Value	r	*p*-Value
Tissue markers						
T2* (ms)	–0.325	**0.007**	–0.244	0.045	0.154	0.209
T1 mapping (ms)	–0.448	**<0.001**	–0.126	0.307	0.283	**0.019**
Biological						
Ferritin (μg/L)	0.080	0.516	0.025	0.840	–0.056	0.651
Hbg (g/dL)	0.047	0.704	0.172	0.161	0.047	0.702
Geometric						
LVM	0.204	0.095	–0.163	**0.041**	–0.149	0.224
LVM/EDV	0.168	0.171	–0.449	**<0.001**	–0.096	0.435

Hbg, hemoglobin; LVM, left ventricle mass; LVM/EDV; r, Pearson’s correlation coefficient. Significant *p*-values are indicated in bold.

**Table 6:** Multivariable linear regression.

Variable	GCS		GLS		GRS	
	β	*p*-Value	β	*p*-Value	β	*p*-Value
Age	0.085	0.736	0.162	0.512	–0.080	0.775
BSA	0.125	0.615	–0.015	0.952	–0.006	0.982
hemoglobin	–0.018	0.884	0.127	0.297	0.078	0.567
LVM	–0.127	0.282	–0.142	0.089	0.014	0.912
sex	0.041	0.737	–0.019	0.872	–0.096	0.478
T2*	–0.281	**0.021**	–0.263	**0.034**	0.054	0.723

*β*: standardized coefficients (beta). Significant *p*-values are indicated in bold.

**Table 7:** Intra-observer and inter-observer variability.

Variable	Intra-Observer	Inter-Observer
GCS	0.99 (0.97–0.99)	0.90 (0.66–0.97)
GLS	0.97 (0.91–0.99)	0.98 (0.92–0.99)
GRS	0.82 (0.76–0.88)	0.91 (0.64–0.97)

Data is represented using intraclass correlation coefficients (95% CI).

## Data Availability

The data presented in this study are available on request from the corresponding author due to privacy restrictions.

## Abbreviations

The following abbreviations are used in this manuscript:

CMR	Cardiovascular Magnetic Resonance
LV	Left Ventricle
FT	Feature Tracking
GCS	Global Circumferential Strain
GLS	Global Longitudinal Strain
GRS	Global Radial Strain
AI	Artificial Intelligence

## References

[1] Sumantri N.I., Pratama H., Davida A., Rizkinia M. (2021). Uncover molecular network between beta thalassemia and retinal abnormality through in silico study. AIP Conf. Proc..

[2] Soteriades E.S., Angastiniotis M., Economidou E.C., Farmakis D., Avraam D., Eleftheriou A. (2025). The disease burden of β-thalassaemia revisited. Hematology.

[3] Li L., Mandal P.K. (2024). Recent advancements in gene therapy for sickle cell disease and β-thalassemia. Front. Hematol..

[4] Sadiq I.Z., Abubakar F.S., Usman H.S., Abdullahi A.D., Ibrahim B., Kastayal B.S., Ibrahim M., Hassan H.A. (2024). Thalassemia: Pathophysiology, Diagnosis, and Advances in Treatment. Thalass. Rep..

[5] Dan M.-O., Gutu B.-I., Severin E., Tanase V.-G. (2023). Innovative and needs-led research on β-thalassemia treatment methods. Front. Hematol..

[6] Sumneang N., Siri-Angkul N., Kumfu S., Chattipakorn S.C., Chattipakorn N. (2020). The effects of iron overload on mitochondrial function, mitochondrial dynamics, and ferroptosis in cardiomyocytes. Arch. Biochem. Biophys..

[7] Wood J.C. (2008). Cardiac iron across different transfusion-dependent diseases. Blood Rev..

[8] Russo V., Melillo E., Papa A.A., Rago A., Chamberland C., Nigro G. (2019). Arrhythmias and sudden cardiac death in beta-thalassemia major patients: Noninvasive diagnostic tools and early markers. Cardiol. Res. Pract..

[9] Hussien M., Bermudez F., Bering P.T., Weissman G., Hays A.G., Sheikh F.H. (2025). Cardiac Magnetic Resonance Imaging in the Evaluation and Prognosis of Infiltrative Cardiomyopathies. J. Cardiovasc. Dev. Dis..

[10] Torlasco C., Cassinerio E., Roghi A., Faini A., Capecchi M., Abdel-Gadir A., Giannattasio C., Parati G., Moon J.C., Cappellini M.D. (2018). Role of T1 Mapping as a Complementary Tool to T2* for Non-Invasive Cardiac Iron Overload Assessment. PLoS ONE.

[11] Kim P.K., Hong Y.J., Im D.J., Suh Y.J., Park C.H., Kim J.Y., Chang S., Lee H.J., Hur J., Kim Y.J. (2017). Myocardial T1 and T2 Mapping: Techniques and Clinical Applications. Korean J. Radiol..

[12] Baxa J., Ferda J., Hromádka M. (2016). T1 Mapping of the Ischemic Myocardium: Review of Potential Clinical Use. Eur. J. Radiol..

[13] Ferreira V.M., Piechnik S.K. (2020). CMR Parametric Mapping as a Tool for Myocardial Tissue Characterization. Korean Circ. J..

[14] Menacho K., Abdel-Gadir A., Moon J.C., Fernandes J.L. (2019). T2* Mapping Techniques: Iron Overload Assessment and Other Potential Clinical Applications. Magn. Reson. Imaging Clin. N. Am..

[15] Bayraktaroğlu S., Aydınok Y., Yıldız D., Uluer H., Savaş R., Alper H. (2011). The Relationship between the Myocardial T2* Value and Left Ventricular Volumetric and Functional Parameters in Thalassemia Major Patients. Diagn. Interv. Radiol..

[16] Daar S., Pathare A.V., Jain R., Zadjali S.A., Pennell D.J. (2009). T2* Cardiovascular Magnetic Resonance in the Management of Thalassemia Patients in Oman. Haematologica.

[17] Ojha V., Ganga K.P., Seth T., Roy A., Naik N., Jagia P., Gulati G.S., Kumar S., Sharma S. (2021). Role of CMR Feature-Tracking Derived Left Ventricular Strain in Predicting Myocardial Iron Overload and Assessing Myocardial Contractile Dysfunction in Patients with Thalassemia Major. Eur. Radiol..

[18] Sobh D.M., Batouty N.M., Tawfik A.M., Gadelhak B., Elmokadem A.H., Hammad A., Eid R., Hamdy N. (2021). Left Ventricular Strain Analysis by Tissue Tracking–Cardiac Magnetic Resonance for Early Detection of Cardiac Dysfunction in Children with End Stage Renal Disease. J. Magn. Reson. Imaging.

[19] Magri D., Sciomer S., Fedele F., Gualdi G., Casciani E., Pugliese P., Losardo A., Ferrazza G., Pasquazzi E., Schifano E. (2008). Early Impairment of Myocardial Function in Young Patients with β-Thalassemia Major. Eur. J. Haematol..

[20] Villegas-Martinez M., De Villedon De Naide V., Muthurangu V., Bustin A. (2024). The Beating Heart: Artificial Intelligence for Cardiovascular Application in the Clinic. Magn. Reson. Mater. Phys. Biol. Med..

[21] Ghadimi S., Auger D.A., Feng X., Sun C., Meyer C.H., Bilchick K.C., Cao J.J., Scott A.D., Oshinski J.N., Ennis D.B. (2021). Fully-Automated Global and Segmental Strain Analysis of DENSE Cardiovascular Magnetic Resonance Using Deep Learning for Segmentation and Phase Unwrapping. J. Cardiovasc. Magn. Reson..

[22] Jiao R., Liu X., Shao S., Zhou K., Zhao L., Pu B., Hua Y., Guo X., Cai X., Zhang L. (2025). Digital Profile of Children’s Hearts: Automated Echocardiogram Strain Analysis Facilitates Earlier Detection of Cardiac Dysfunction. Eur. Heart J..

[23] Abramikas Ž., Jasiukevičiūtė I., Balčiūnaitė G., Glaveckaitė S., Palionis D., Valevičienė N. (2025). Artificial Intelligence Performance in Cardiac Magnetic Resonance Strain Analysis for Aortic Stenosis: Validation with Echocardiography and Healthy Controls. Medicina.

[24] Feng Y., He T., Carpenter J.P., Jabbour A., Alam M.H., Gatehouse P.D., Greiser A., Messroghli D., Firmin D.N., Pennell D.J. (2013). In Vivo Comparison of Myocardial T1 with T2 and T2* in Thalassemia Major. J. Magn. Reson. Imaging.

[25] Claus P., Omar A.M.S., Pedrizzetti G., Sengupta P.P., Nagel E. (2015). Tissue Tracking Technology for Assessing Cardiac Mechanics: Principles, Normal Values, and Clinical Applications. JACC Cardiovasc. Imaging.

[26] Xu H.Y., Chen J., Yang Z.G., Li R., Shi K., Zhang Q., Liu X., Xie L.J., Jiang L., Guo Y.K. (2017). Early Marker of Regional Left Ventricular Deformation in Patients with Hypertrophic Cardiomyopathy Evaluated by MRI Tissue Tracking: The Effects of Myocardial Hypertrophy and Fibrosis. J. Magn. Reson. Imaging.

[27] Zhang Z., Ma Q., Cao L., Zhao Z., Zhao J., Lu Q., Zeng L., Zhang M., Pohost G.M., Li K. (2019). Correlation between Left Ventricular Myocardial Strain and Left Ventricular Geometry in Healthy Adults: A Cardiovascular Magnetic Resonance–Feature 608 Tracking Study. Int. J. Cardiovasc. Imaging.

[28] Rezaeian N., Mohtasham M.A., Khaleel A.J., Parnianfard N., Kasani K., Golshan R. (2020). Comparison of Global Strain Values of Myocardium in Beta-Thalassemia Major Patients with Iron Load Using Specific Feature Tracking in Cardiac Magnetic Resonance Imaging. Int. J. Cardiovasc. Imaging.

[29] Parsaee M., Akiash N., Azarkeivan A., Alizadeh Sani Z., Amin A., Pazoki M., Samiei N., Jalili M.A., Adel M.H., Rezaian N. (2018). The Correlation between Cardiac Magnetic Resonance T2* and Left Ventricular Global Longitudinal Strain in People with β-Thalassemia. Echocardiography.

[30] Meloni A., Saba L., Positano V., Pistoia L., Campanella A., Spasiano A., Putti M.C., Fotzi I., Cossu A., Corigliano E. (2024). Global Longitudinal Strain by Cardiac Magnetic Resonance Is Associated with Cardiac Iron and Complications in Beta-Thalassemia Major Patients. Int. J. Cardiol..

[31] Ansah D., Husain N., Ruh A., Berhane H., Smith A., Thompson A., De Freitas A., Rigsby C.K., Robinson J.D. (2023). Cardiac Magnetic Resonance Strain in Beta Thalassemia Major Correlates with Cardiac Iron Overload. Children.

[32] Triadyaksa P., Oudkerk M., Sijens P.E. (2020). Cardiac T2* Mapping: Techniques and Clinical Applications. J. Magn. Reson. Imaging.

[33] Saliba A.N., Atoui A., Labban M., Hamade H., Bou-Fakhredin R., Mufarrij A., Taher A.T. (2020). Thalassemia in the emergency department: Special considerations for a rare disease. Ann. Hematol..

[34] Meloni A., Saba L., Cademartiri F., Positano V., Pistoia L., Cau R. (2024). Cardiovascular Magnetic Resonance in β-Thalassemia Major: Beyond T2*. Radiol. Med..

[35] Musallam K.M., Cappellini M.D., Taher A.T. (2013). Iron Overload in β-Thalassemia Intermedia: An Emerging Concern. Curr. Opin. Hematol..

[36] Khaled A., Ezzat D.A., Salem H.A., Seif H.M., Rabee H. (2019). Effective Method of Evaluating Myocardial Iron Concentration in Pediatric Patients with Thalassemia Major. J. Blood Med..

[37] Darvishi-Khezri H., Aliasgharian A., Naderisorki M., Kosaryan M., Ghazaiean M., Fallah H., Zahedi M., Karami H. (2022). Ferritin thresholds for cardiac and liver hemosiderosis in β-thalassemia patients: A diagnostic accuracy study. Sci. Rep..

[38] Awadi R., Benameur N., Hafsi H., Younes T.B., Arous Y., Labidi S., Tavares J.M.R.S. (2024). Myocardial Strain Assessment for Early Duchenne Muscular Dystrophy Diagnosis in Pediatric Patients Using Cardiac MRI. Appl. Sci..

[39] Mizuguchi Y., Oishi Y., Miyoshi H., Iuchi A., Nagase N., Oki T. (2010). Concentric left ventricular hypertrophy brings deterioration 626 of systolic longitudinal, circumferential, and radial myocardial deformation in hypertensive patients with preserved left ven-627 tricular pump function. J. Cardiol..

[40] Pryds K., Larsen A.H., Hansen M.S., Grøndal A.Y.K., Tougaard R.S., Hansson N.H., Clemmensen T.S., Løgstrup B.B., Wiggers H., Kim W.Y. (2019). Myocardial Strain Assessed by Feature Tracking Cardiac Magnetic Resonance in Patients with a Variety of Cardiovascular Diseases—A Comparison with Echocardiography. Sci. Rep..

